# Total extract of *Abelmoschus manihot* L. alleviates uric acid-induced renal tubular epithelial injury *via* inhibition of caspase-8/caspase-3/NLRP3/GSDME signaling

**DOI:** 10.3389/fphar.2022.907980

**Published:** 2022-08-16

**Authors:** Zhihui Ding, Jing Zhao, Xufang Wang, Wei Li, Chong Chen, Chen Yong, Yiye Zhu, Fang Tian, Li Liu, Manshu Yu, Enchao Zhou, Liubao Gu, Chunlei Yao, Kun Gao

**Affiliations:** ^1^ Division of Nephrology, Affiliated Hospital of Nanjing University of Chinese Medicine, Nanjing, China; ^2^ Division of Nephrology, Taizhou Second People’s Hospital, Taizhou, China; ^3^ Division of Clinical Epidemiology, Geriatric Hospital of Nanjing Medical University, Nanjing, China

**Keywords:** pyroptosis, uric acid, caspase-8, caspase-3, NLRP3, GSDME, total extract of Abelmoschus manihot L. flower

## Abstract

**Purpose:** The incidence of uric acid (UA)-induced kidney injury is increasing owing to the high incidence of hyperuricemia in recent years. The flower of *Abelmoschus manihot* (Linneus) Medik is a traditional Chinese medicinal herb widely used in the treatment of some kidney diseases. In our previous study, we reported that the total extract of *A. manihot* L. flower (TEA) attenuated adriamycin-induced renal tubular cell injury. In this study, we aimed to evaluate the role of TEA in UA-induced tubular cell injury.

**Methods:** Normal rat proximal epithelial NRK-52E cells were incubated with UA to mimic hyperuricemia conditions. The role of TEA in the renal tubular cells was also assessed. The cellular morphology was observed using phase-contrast microscopy, and cell viability was analyzed using the Cell Counting kit-8. Living and dead cells were stained using a Calcein-AM/PI double stain kit. The release of lactate dehydrogenase (LDH) was analyzed by LDH cytotoxicity Assay Kit. The expression of target proteins was analyzed using western blot analysis.

**Results:** UA triggered NRK-52E cell injury, as evidenced by morphological changes, detachment of cells from the bottom, cell swelling, large bubbles blowing from cell membrane and loss of cell viability. UA increased release of LDH. UA induced the expression of p-ERK1/2 and the subsequent activation of caspase-8, caspase-3, and NLRP3 inflammasomes. Pyroptosis was elicited by UA after gasdermin E N-terminal (GSDME-NT) was cleaved from gasdermin E (GSDME). Z-DEVD-FMK, a caspase-3 inhibitor, suppressed the expression of both NLRP3 and GSDME-NT, but not that of caspase-8. INF39, an NLRP3 inhibitor, altered the expression of GSDME-NT expression, but not that caspase-3 and caspase-8. TEA alleviated UA-induced cell injury by suppressing ERK1/2/caspase-8/caspase-3/NLRP3/GSDME signaling.

**Conclusion:** GSDME-mediated pyroptosis was involved in UA-induced renal tubular cell injury. This is the first study to report that TEA protects renal tubular epithelial cells against UA by inhibiting the ERK/1/2/caspase-8/caspase-3/NLRP3/GSDME pathway.

## Introduction

The incidence of hyperuricemia is gradually increasing with lifestyle changes. The overall prevalence of hyperuricemia among adults in the United States was 20.1% between 2015-2016 (Chen-Xu et al., 2019). In China, the pooled prevalence of hyperuricemia was 13.3% between 2000-2014 (Liu et al., 2015). Recent reports have confirmed that hyperuricemia is associated with cardiovascular and renal diseases. Serum uric acid (UA) is a major predictor of the development of kidney diseases, and its increased level is associated with decreased kidney function (Domrongkitchaiporn et al., 2005; Obermayr et al., 2008; Rosolowsky et al., 2008; Bellomo et al., 2010). Nephrologists are currently faced with the challenges of dissecting the molecular mechanism underlying UA-induced renal damage and exploring new therapeutic drugs.

UA, which is the end product of purine-derivative metabolism, has dual roles, including antioxidative and pro-oxidative effects (Kang and Ha, 2014). The anti-oxidative effect is observed at normal UA concentrations, whereas UA exerts pro-oxidative function at high concentrations. Studies have shown that UA induces renal tubular epithelial injury by promoting oxidative stress (Verzola et al., 2014; Li et al., 2016; Yang et al., 2019) or inflammation (Zhou Y. et al., 2012; An et al., 2014; Braga et al., 2020; Wu et al., 2021). Pyroptosis (Rashidi et al., 2019; Shen et al., 2021) and apoptosis (Verzola et al., 2014; Yang et al., 2019; Li D. et al., 2021) are involved in UA-induced cell death. However, the mechanism of pyroptosis underlying UA cytotoxicity is not fully understood.

Pyroptosis is a type of gasdermin-mediated programmed inflammatory necrotic cell death (Shi et al., 2017). The canonical pathway of pyroptosis involves NOD-like receptors (NLRs) that recognize stimulus signals, reassemble the NLR family pyrin domain-containing 3 (NLRP3) inflammasome, activate caspase-1, cleave gasdermin D (GSDMD) to form GSDMD-N terminal, generate membrane pores, and release proinflammatory cytokines, resulting in cell swelling and eventual lysis (Shi et al., 2017; Wang et al., 2021). Recent studies have reported the involvement of both gasdermin E (GSDME) and caspase-3 in renal pyroptosis, including in diabetic nephropathy and obstructive nephropathy (Li W. et al., 2021; Li Y. et al., 2021). Caspase-8, a typical cysteine protease, induces pyroptosis by cleaving GSDMD and GSDME (Orning et al., 2018; Sarhan et al., 2018). Caspases are important upstream regulators of NLRP3; however, the detailed role of specific caspases in pyroptosis has not been fully studied.

The flower of *Abelmoschus manihot* (Linneus) Medik. is a traditional Chinese herb with a long history of treating chronic kidney disease (CKD) in China. Compounds of the flower of *A. manihot* (Linneus) Medik. have been isolated and purified using chromatographic techniques. Their structures were identified by analyzing physicochemical properties and spectral data, as described in our previous reports (Li et al., 2019). Several studies have confirmed the renal protective properties of *A. manihot* (Chen et al., 2016). A prospective, multicenter randomized controlled clinical trial (RCT) confirmed that *A. manihot* could lower proteinuria in patients with CKD stages 1-2 (Zhang et al., 2014). Further studies suggest that *A. manihot* is more effective than losartan in reducing proteinuria in patients with primary glomerular disease (Carney, 2014). *A. manihot* prevents podocyte apoptosis in streptozotocin-induced (STZ) diabetic nephropathy (DN) by inhibiting caspase-3 and caspase-8 expressions (Zhou L. et al., 2012). Our previous studies reported that the total extract of *A. manihot* L. (TEA) attenuates renal tubular cell oxidative injury (Zhou L. et al., 2012; Li et al., 2019). In traditional Chinese medicine (TCM), *A. manihot* has also been used to treat hyperuricemia. However, the role of *A. manihot* in UA-induced renal tubular epithelial cell injury remains unclear.

Thus, in the current study, we employed an *in vitro* model of UA-induced renal tubular epithelial cell injury to investigate the role of pyroptosis and explore the possible mechanisms underlying the effect of TEA on this type of injury.

## Materials and methods

### Cell culture

Normal rat proximal epithelial cell line, NRK-52E, was obtained from the University of Yamanashi (Yamanashi, Japan) and cultured in Dulbecco’s modified Eagle’s medium/F-12 (DMEM/F12, Gibco, United States) containing 100 U/mL penicillin G, 100 mg/ml streptomycin (Gibco, United States), and 5% fetal bovine serum (FBS, Gibco, United States). The NRK-52E cells were incubated at 37°C in an incubator with 5% CO_2_. All experiments were performed after the cells were seeded in a medium containing 1% FBS for 24 h.

### Preparation of total extract of *A. manihot* L

TEA was extracted by the Department of Drug Preparation of the Affiliated Hospital of Nanjing University of Chinese Medicine. TEA was prepared as described in our previous study (Li et al., 2019). In brief, 500 g of raw *A. manihot* (Linneus) Medik flowers was soaked in 8,000 ml of 75% ethanol for 1 h; then the mixture was warmed to 90°C and maintained at that temperature for another 1 h to allow alcohol extraction of the ambrette fluid. After filtration, the ambrette fluid extract was evaporated to obtain a dry extract powder under vacuum at 60°C. The dried residue was dissolved in water for subsequent experiments. The profile composition of TEA was characterized using high-performance liquid chromatography (HPLC). The HPLC profile of TEA was shown in Supplementary Material S1. TEA was composed of the following compounds: Hyperoside (43.2%), hibifolin (27.1%), isoquercetin (13.7%), Quercetin-3′-O-glucoside (8.8%), quercetin-3-O-robinobioside (3.8%), myricetin (3.2%), and quercetin (0.2%) (Zhou L. et al., 2012).

### Reagents and antibodies

Primary antibodies against phospho-ERK (lot no. 4370, CST), caspase-3 (lot no. 14220, CST), and caspase-8 (lot no. 4790) were purchased from Cell Signaling Technology (CST) Shanghai Biological Reagents Co., Ltd. (Shanghai, China). Primary antibodies against NLRP3 (lot no. ab263899, Abcam) and DFNA5/GSDME (lot no. ab215191; Abcam) were purchased from Abcam (Cambridge, UK). INF 39 (NLRP3 inhibitor, HY-101868) was purchased from MedChemExpress (NJ, United States). Z-DEVD-FMK (caspase-3 inhibitor, RM02811) was purchased from ABclonal Technology Co., Ltd. (Hubei, China). Primary antibody against β-tubulin (lot no. T0023; Affinity Biosciences) was purchased from Affinity Biosciences (Jiangsu, CN). Primary antibody against β-actin (lot no.66009-I-Ig, Proteintech) was purchased from Proteintech (Chicago, United States). Horseradish peroxidase-conjugated anti-rabbit IgG and horseradish peroxidase-conjugated anti-mouse IgG were purchased from Biosharp (Shanghai, China). UA was purchased from Macklin (Shanghai, China).

### Assessment of cell viability with Cell Counting Kit-8

Cells were seeded in 96-well plates and exposed to specific stimuli. CCK-8 reagent (lot no. K10189133EF5E, APEXBIO) was added to each well, and cells were incubated at 37°C in an incubator with 5% CO_2_ for 1 h. The absorbance was measured at 450 nm using a microplate reader (ELX-800, BIOTEK). Cell viability was expressed as the percentage of control cells.

### Western blot analysis

Protein concentrations were determined using the BCA (bicinchoninic acid) Protein Assay Kit (Beyotime Biotechnology, China), following the manufacturer’s instructions. Equal amounts of protein were added to sodium dodecyl sulfate-polyacrylamide (SDS-PAGE) gels, separated by electrophoresis, and then transferred to polyvinylidene difluoride (PVDF) membranes. The PVDF membranes were incubated with the primary antibody overnight at 4°C after blocking with 5% non-fat dried milk in phosphate-buffered saline (PBS) containing 0.2% Tween-20 (PBST) for 1 h at room temperature. After washing with PBST, membranes were incubated with horseradish peroxidase-conjugated anti-rabbit or anti-rat IgG for 1 h at room temperature. The bands were scanned using a ChemiDoc XRS + Imaging System (Bio-Rad, United States). The scanned signal was quantitatively analyzed using the ImageJ software (1.8.0). β-actin or β-tubulin was used as loading controls.

### Calcein-AM/PI double staining

Dual staining of live and dead cells was performed using the Calcein-AM/PI double stain kit (lot no. 40747ES76; Yeasen Biotechnology Ltd. [Shanghai, China]), following the manufacturer’s instructions. NRK-52E cells were seeded in 96-well plates and treated with UA or TEA for 24 h. The cells were then incubated with a dyeing working fluid consisting of 2 μM calcein-AM and 4.5 μM PI for 15 min at 37°C. Cells were visualized and captured under an inverted fluorescence microscope (ECLIPSE Ts2R; Nikon, Japan). ImageJ software (version 1.8.0) was used to count the percentage of fluorescent cells.

### The release of lactate dehydrogenase (LDH) assay

Cells were seeded in 96-well plates and exposed to specific stimuli. LDH reagent (lot no. C0016, Beyotime) was added to cell culture in supernatant of each well, and were incubated avoid light at 25°C for 30 min. The absorbance was measured at 490 nm using a microplate reader (ELX-800, BIOTEK). The release of LDH was expressed as the percentage of control group.

### Statistical analysis

The results are expressed as the mean ± standard deviation. Differences among groups were analyzed using Student’s t-test or one-way analysis of variance. Data were analyzed using the SPSS software (version 23.0; IBM Corp. NY, United States). Values with *p* < 0.05 were considered statistically significant.

## Results

### UA triggers renal tubular cell injury and induces cell death

**FIGURE 1:**
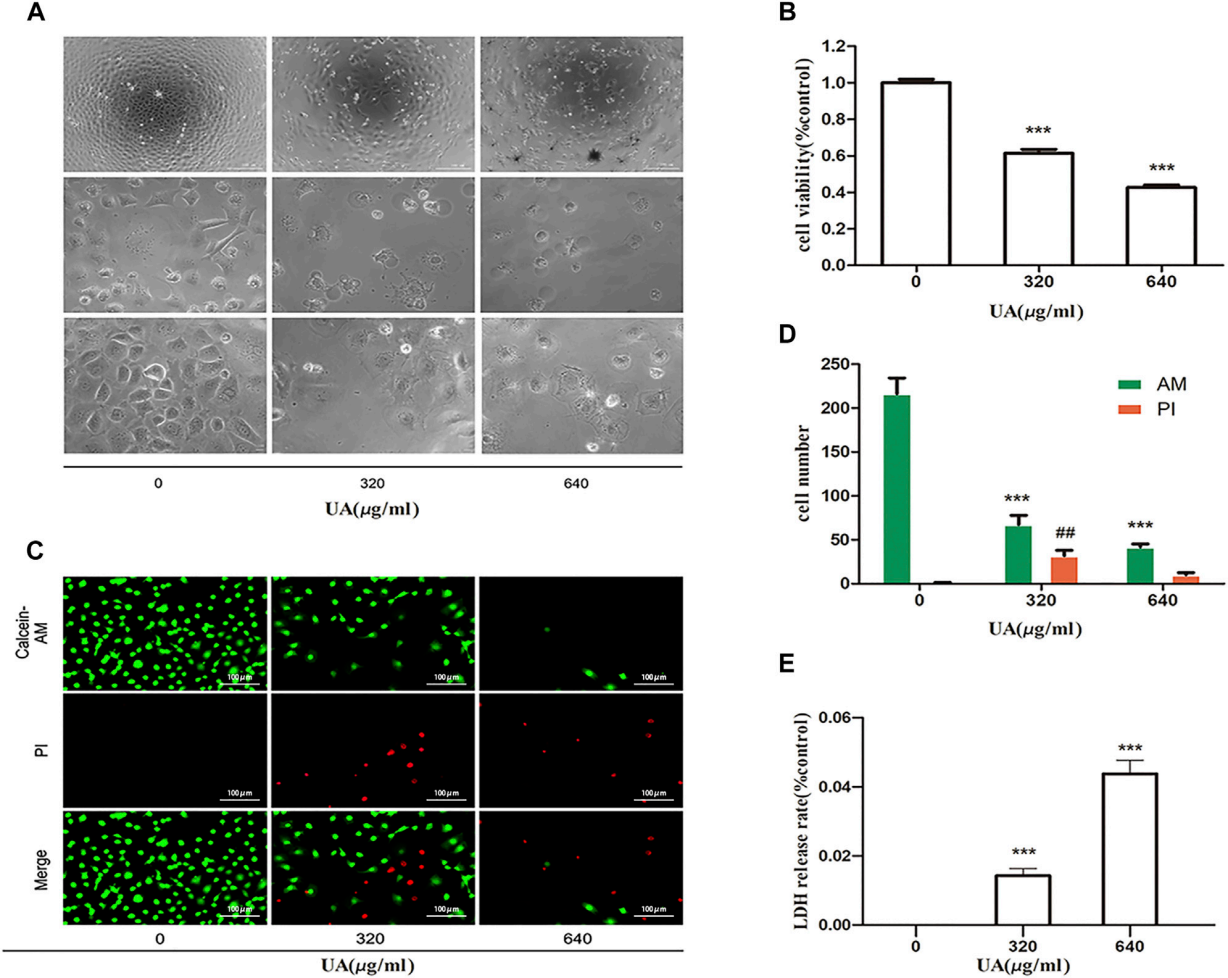
Uric acid (UA)-induced NRK-52E cell injury. **(A)** Effect of UA on morphological changes. NRK-52E cells were treated with different concentrations of UA (0, 320, and 640 μg/ml) for 24 h. Cell morphology was analyzed using phase-contrast microscopy (magnification,×100, ×400). **(B)** Effects of UA on cell viability. NRK-52E cells in 96-well plates were exposed to different concentrations (0, 320, and 640 μg/ml) of UA for 24 h. Cell viability was evaluated using a CCK-8 assay. Data are expressed as the percentages of living cells versus the control group (0 μg/ml) (means ± SD, n = 5 in each group). ∗∗∗*p* < 0.001 versus the control group. **(C)** Living and dead cells were stained with calcein-AM or PI and then visualized by inverted fluorescence microscopy (magnification, ×200). The cells emitting green fluorescence were living cells, and those emitting red fluorescence were dead cells. **(D)** The number of different cells was calculated by ImageJ software. Data are expressed as the number of positively stained cells. Experiments were performed three times. ∗∗*p* < 0.01 or ##*p* < 0.01 versus control (0 μg/ml) group. **(E)** The LDH release rate of UA on cells. NRK-52E cells in 96-well plates were exposed to different concentrations (0, 320, and 640 μg/ml) of UA for 24 h. LDH release rate was analyzed by LDH cytotoxicity Assay Kit. All data were corrected with respect to the control group (0 μg/ml) (means ± SD, *n* = 6 in each group). ∗∗∗*p* < 0.001 versus the control group.

First, we confirmed the cytotoxic effects of UA in NRK-52E cells. As shown in Figures 1A,B, after incubation with different concentrations of UA for 24 h, the cells showed morphological changes (cell swelling, large bubbles blowing from cell membrane), loss of cell-to-cell contact, and detachment from the bottom of the dish in a dose-dependent manner. Meanwhile, cell viability was lost after treatment of cells with UA at 640 μg/ml concentration, which was determined as the concentration of the subsequent experiment (Figure 1B). As shown in Figures 1C,D, after treatment with UA, the number of living cells was reduced, as visualized by using calcein-AM/PI double staining. The release of LDH from UA treated cells was greatly increased in a dose-dependent manner (Figure 1E). These results suggest that high UA concentrations trigger renal tubular cell injury and death.

### TEA ameliorates UA-induced renal tubular cell injury

**FIGURE 2:**
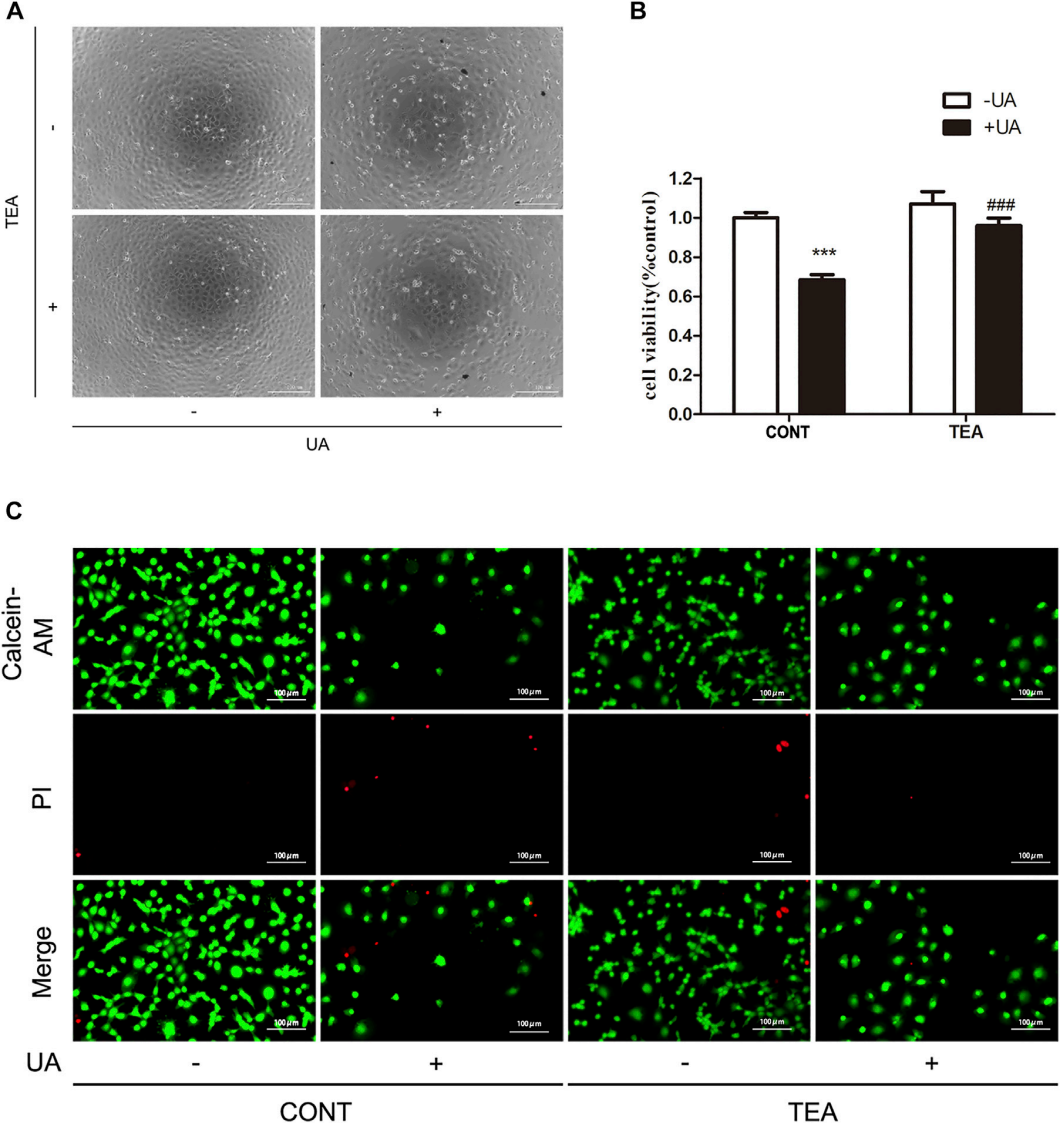
*Abelmoschus manihot* L. flower (TEA) can alleviate uric acid (UA)-induced NRK-52E cell injury. **(A)** Effect of UA and TEA on morphological changes. NRK-52E cells were treated with UA and TEA for 24 h. Cell morphology was analyzed using phase-contrast microscopy (magnification, ×100). **(B)** Effects of TEA on cell viability. NRK-52E cells in 96-well plates were treated with TEA and UA for 24 h. Cell viability was evaluated using a CCK-8 assay. Data are expressed as the percentages of living cells versus the control group (CONT, 0 μg/ml) (means ± SD, n = 3 in each group). ∗∗∗*p* < 0.001 versus the CONT group; or ###*p* < 0.001 versus UA group in CONT. **(C)** Living and dead cells were stained with calcein-AM or PI and then visualized by inverted fluorescence microscopy (magnification, ×200). The cells emitting green fluorescence were living cells, and those emitting red fluorescence were dead cells.

Next, we sought to determine if TEA could attenuate UA-induced cell injury. As shown in Figure 2A, TEA restored the cellular morphological changes induced by UA. Furthermore, TEA ameliorated the UA-induced decrease in cell viability (Figure 2B). Calcein-AM/PI double staining showed similar results (Figure 2C). These results show that TEA ameliorates UA-induced renal tubular cell injury. However, the exact mechanism by which TEA ameliorates UA-induced renal tubular cell injury remains to be elucidated.

### TEA attenuates UA-induced pyroptosis by inhibiting expression of GSDME-NT, NLRP3, and caspases

**FIGURE 3:**
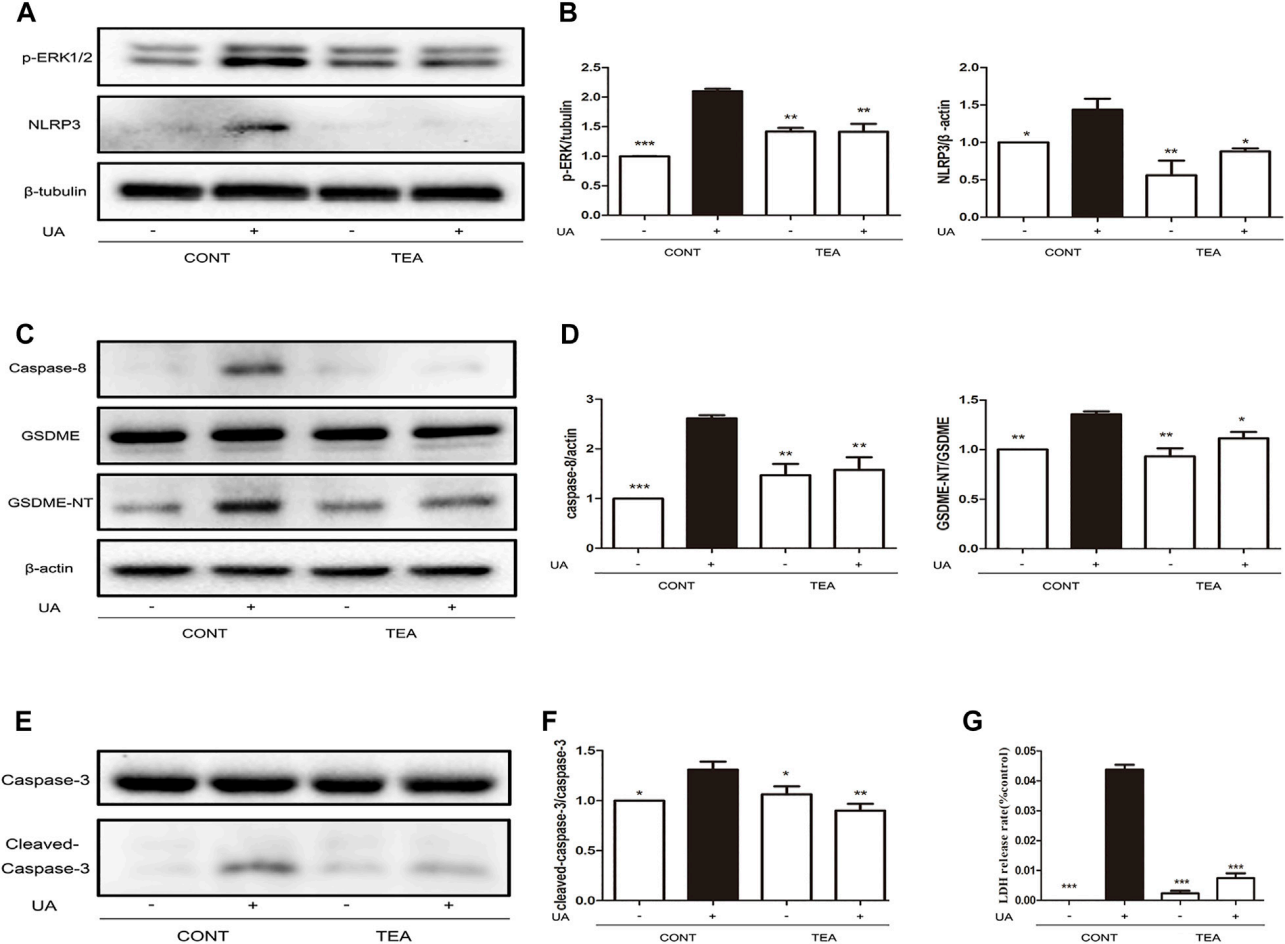
*Abelmoschus manihot* L. flower (TEA) attenuates uric acid (UA)-induced pyroptosis by inhibiting the expression of GSDME-NT, NLRP3, and caspases. **(A)** Effects of TEA on ERK1/2 phosphorylation and NLRP3 expression induced by UA. NRK-52E cells in 6-well plates were treated with TEA and UA for 24 h. **(B)** Statistical analyses of ERK1/2 phosphorylation and NLRP3 expression (means ± SD, *n* = 3 in each group). All data were corrected with respect to the control group. ∗∗∗*p* < 0.001 versus the model group (UA in 640 μg/ml); ∗∗*p* < 0.01 versus the model group (UA in 640 μg/ml); ∗*p* < 0.05 versus the model group (UA in 640 μg/ml). **(C)** Effects of TEA on caspase-8 and GSDME expression induced by UA. NRK-52E cells in 6-well plates were treated with TEA and UA for 24 h. **(D)** Statistical analyses of caspase-8 and GSDME expression (means ± SD, *n* = 3 in each group). All data were corrected with respect to the control group. ∗∗∗*p* < 0.001 versus the model group (UA in 640 μg/ml); ∗∗*p* < 0.01 versus the model group (UA in 640 μg/ml); ∗*p* < 0.05 versus the model group (UA in 640 μg/ml). **(E)** Effects of TEA on caspase-3 expression induced by UA. NRK-52E cells in 6-well plates were treated with TEA and UA for 24 h. **(F)** Statistical analyses of caspase-3 expression (means ± SD, *n* = 3 in each group). All data were corrected with respect to the control group. ∗∗∗*p* < 0.001 versus the model group (UA in 640 μg/ml); ∗∗*p* < 0.01 versus the model group (UA in 640 μg/ml); ∗*p* < 0.05 versus the model group (UA in 640 μg/ml). **(G)** The LDH release rate of UA and TEA on cells. NRK-52E cells in 96-well plates were treated with UA and TEA for 24 h. LDH release rate was analyzed by LDH cytotoxicity Assay Kit. All data were corrected with respect to the control group (0 μg/ml) (means ± SD, *n* = 6 in each group). ∗∗∗*p* < 0.001 versus the model group (UA in 640 μg/ml).

We previously reported that TEA inhibits expression of ERK1/2-NLRP3 in Adriamycin-induced renal tubular cell injury (Li et al., 2019). Therefore, we explored that whether ERK1/2-NLRP3 signaling is also involved in TEA attenuates UA-induced injury. As indicated by the western blot analysis, TEA suppressed the UA-induced increase in ERK1/2 and NLRP3 expression (Figure 3A). Pyroptosis occurs downstream of NLRP3 activation. Caspase-8 is the inducer and gasdermin E (GSDME) is an executor of pyroptosis (Sarhan et al., 2018). Therefore, we evaluated the occurrence of pyroptosis during UA-induced cell injury. Notably, the UA-induced increase in expression of both caspase-8 and GSDME-N terminal (GSDME-NT) was simultaneously inhibited by TEA (Figures 3C,D). Interestingly, caspase-3 was also cleaved by UA (Figures 3E,F). Furthermore, TEA inhibited UA-induced activation of caspase-3, indicating that TEA attenuated UA-induced pyroptosis by inhibiting the expression of caspase-8, caspase-3, NLRP3, and GSDME-NT. However, whether the expression of caspases is associated with NLRP3 expression remains to be investigated. Moreover, TEA significantly reduced the release of LDH induced by UA (Figure 3G).

### UA induces pyroptosis through the caspase-8/caspase-3/NLRP3/GSDME pathway

**FIGURE 4:**
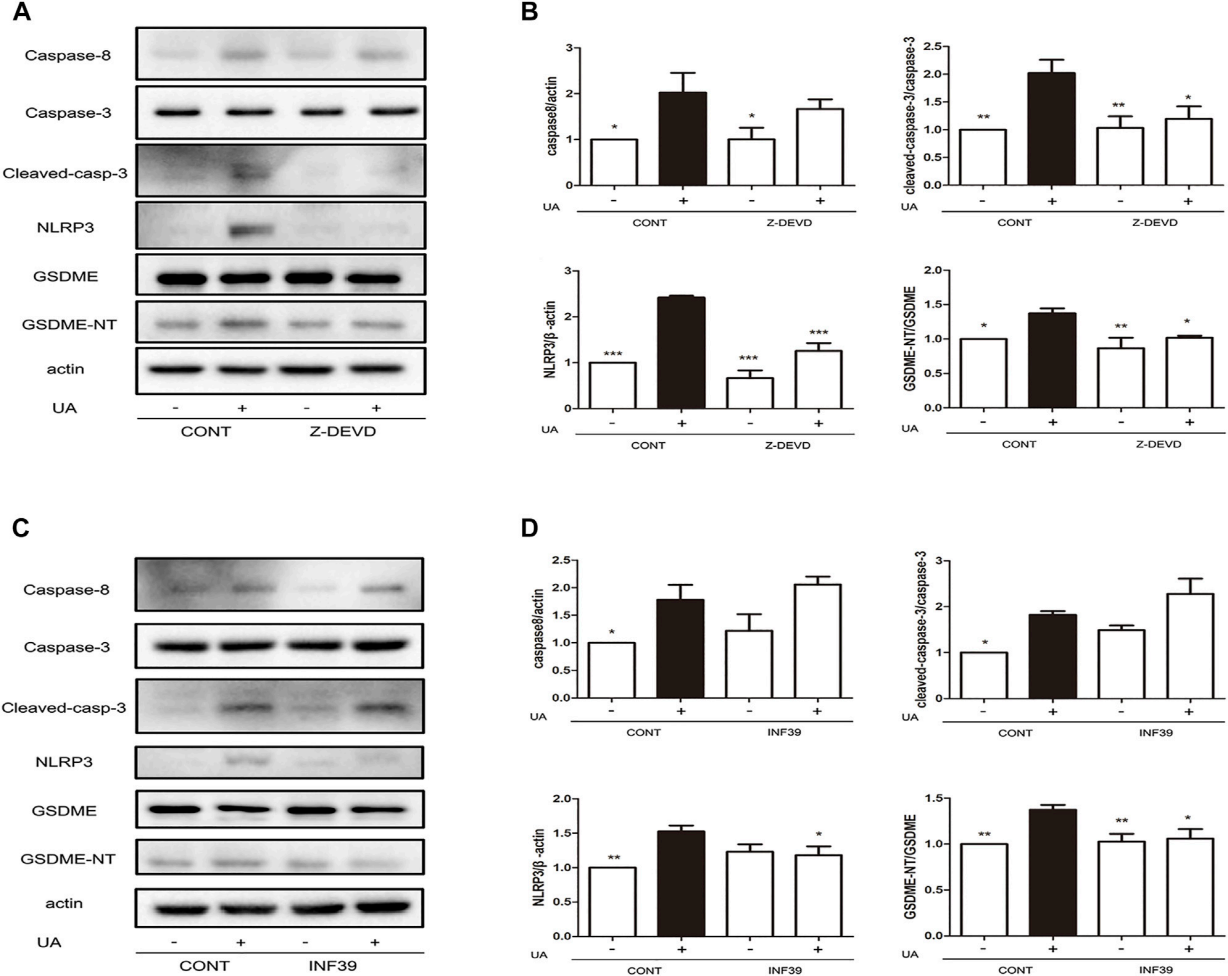
Application of Z-DEVD-FMK and INF39 suppressed caspase-3 and NLRP3 expression. **(A)** Effects of Z-DEVD-FMK on NLRP3, GSDME and caspase-3 expression induced by UA. NRK-52E cells in 6-well plates were treated with Z-DEVD-FMK and UA for 24 h. **(B)** Statistical analyses of caspase-8, caspase-3, NLRP3, and GSDME expression (means ± SD, n = 3 in each group) after treatment of cells with Z-DEVD-FMK and UA. All data were corrected with respect to the control group. ∗∗*p* < 0.01 versus the model group (UA in 640 μg/ml); ∗*p* < 0.05 versus the model group (UA in 640 μg/ml). **(C)** Effects of INF39 on NLRP3, GSDME, and caspase-3 expression induced by UA. NRK-52E cells in 6-well plates were treated with INF39 and UA for 24 h. **(D)** Statistical analyses of caspase-8, caspase-3, NLRP3, and GSDME (means ± SD, *n* = 3 in each group) after treatment of cells with INF39 and UA. All data were corrected with respect to the control group. ∗∗∗*p* < 0.001 versus the model group (UA in 640 μg/ml); ∗∗*p* < 0.01 versus the model group (UA in 640 μg/ml); ∗*p* < 0.05 versus the model group (UA in 640 μg/ml).

To further investigate the regulatory mechanism of UA-induced pyroptosis, we evaluated the association between caspase-3 and NLRP3. UA-induced NRK-52E cells were treated with the specific caspase-3 inhibitor (Z-DEVD-FMK) and NLRP3 inhibitor (INF39). Since cleave of GSDME is regulated by caspase-8 (Sarhan et al., 2018), we investigated the role of caspase-8 in UA-induced pyroptosis. As expected, blocking cleaved caspase-3 expression by Z-DEVD-FMK led to a significant downregulation of NLRP3 (*p* < 0.001) and GSDME-NT expression (Figures 4A,B, *p* < 0.05). Interestingly, caspase-3 upregulation did not affect caspase-8 expression. Furthermore, blocking NLRP3 expression by INF39 led to a significant downregulation of GSDME-NT (*p* < 0.05) but did not disturb the expression of cleaved caspase-3 and caspase-8 (Figures 4C,D). Thus, caspase-8 acts upstream of caspase-3, and both caspase-8 and caspase-3 are involved in UA-induced pyroptosis. Thus, UA induces pyroptosis through the caspase-8/caspase-3/NLRP3/GSDME pathway.

## Discussion

**FIGURE 5:**
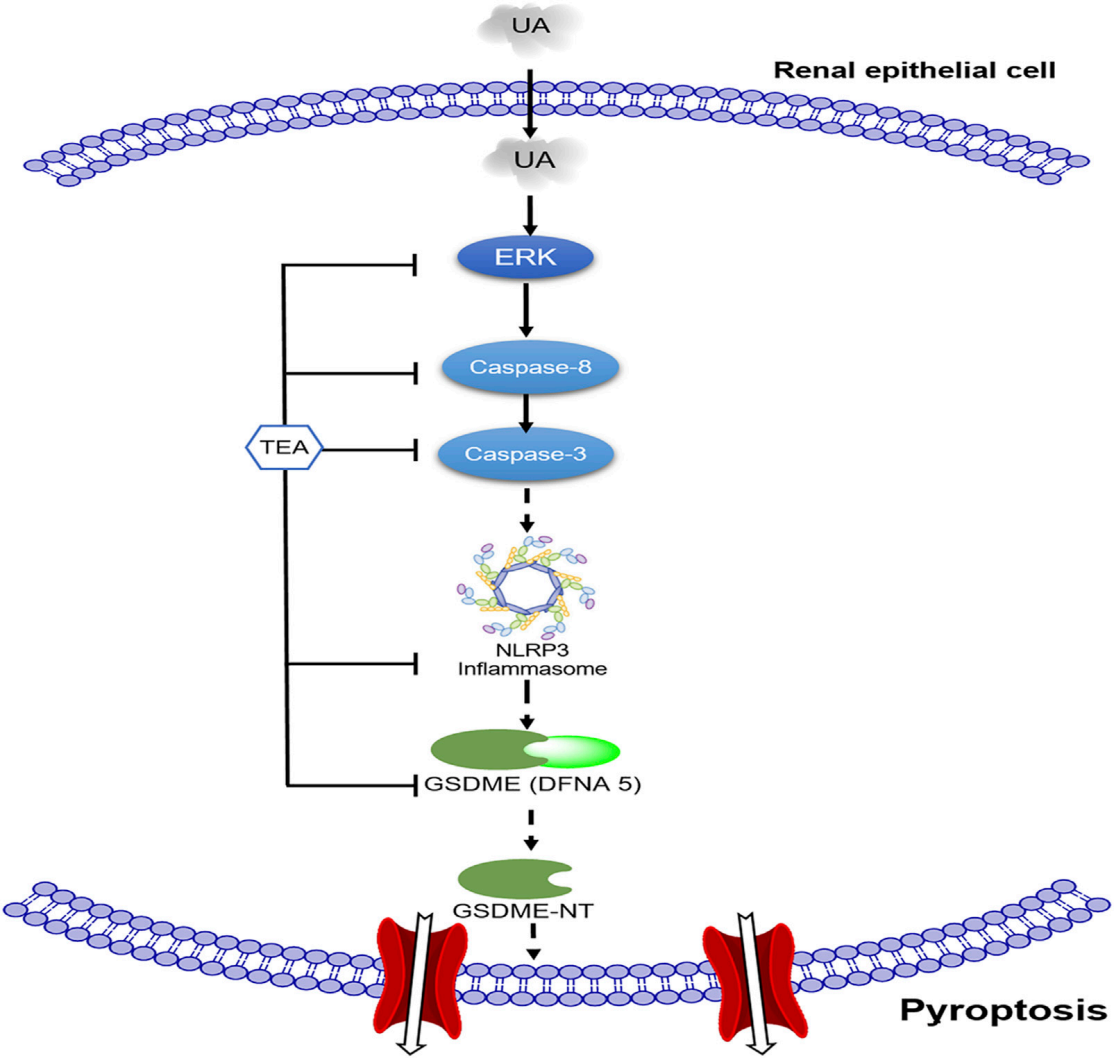
Schematic of the mechanism by which TEA alleviates uric acid (UA)-induced renal tubular epithelial cell injury. First, UA activates ERK1/2 phosphorylation and caspase-8. Subsequently, activated caspase-3 and NLRP3 participate in GSDME-mediated pyroptosis. TEA alleviates UA-induced cell injury through the ERK1/2/caspase-8/caspase-3/NLRP3/GSDME signaling pathway.

The present study confirmed the role of pyroptosis in UA-induced renal tubular epithelial cell injury and explored the association between caspase-8, caspase-3, and NLRP3. The present study is the first to report that TEA attenuates UA-induced pyroptosis by suppressing the caspase-8/caspase-3/NLRP3/GSDME pathway (Figure 5).

Pyroptosis is traditionally defined as programmed inflammatory cell death characterized by gasdermin-dependent membrane pore formation and inflammatory factor release (Galluzzi et al., 2018). The canonical characteristic of pyroptosis is the activation of the pore-formation protein GSDMD (Xu et al., 2018). GSDME, which is expressed in most normal tissues, is also involved in pyroptosis. However, in the present study, GSDMD did not participate in UA-induced cell injury (data not shown). Therefore, we focused only on GSDME. Because of the increase in GSDME-NT triggered by UA, we confirmed that UA elicited GSDME-mediated pyroptosis. Caspase-8/caspase-3 acts upstream of GSDME (Sarhan et al., 2018; Jiang et al., 2020) and can cleave GSDME to generate GSDME-NT, which forms pores in the membrane. Recently, researchers have observed that caspase-3/GSDME-mediated pyroptosis causes ureteral obstruction-induced renal tubule injury (Li Y. et al., 2021) and diabetic nephropathy (Li W. et al., 2021). Studies have also found that GSDME may convert apoptosis into pyroptosis (Rogers et al., 2017; Jiang et al., 2020) in tumors. However, there have been no reports on the involvement of GSDME in UA-induced cell injury. Our findings demonstrate that UA can induce renal tubular epithelial cell pyroptosis through activation of caspase-8 and subsequently caspase-3 to cleave GSDME to form GSDME-N terminal (GSDME-NT). Additionally, TEA can interfere with this process by inhibiting caspase-8/caspase-3/NLRP3/GSDME-NT signal transduction. Our study reveals an important role for caspase-8 and caspase-3 in NLRP3-mediated pyroptosis. NLRP3 participates in the progression of various inflammatory diseases, including gout and arthritis (Wang et al., 2020). Additionally, caspases are one of the most important upstream regulators of NLRP3. The canonical activation of pyroptosis depends on NLRP3/ASC/caspase-1 inflammasome activation to cleave GSDMD (Huang et al., 2021). Some reports suggest that caspase-3 or caspase-8 activates NLRP3 and cleaves the pore formation protein GSDME (Chi et al., 2014; Sarhan et al., 2018; Vince et al., 2018; Zeng et al., 2019). However, it is not clear how upstream molecules of NLRP3 regulate the activation of GSDME in UA-induced cell injury. The present study’s results were consistent with reports demonstrating that UA upregulates NLRP3 (Cabău et al., 2019; Yin et al., 2020; Li D. et al., 2021). We used NLRP3 and caspase-3 specific inhibitors to explore the relationship between NLRP3 and caspase-8/caspase-3/GSDME in UA-induced pyroptosis. Our data indicate that blocking the expression of NLRP3 disturbs the expression of GSDME-NT, but not caspase-3 and caspase-8. In addition, blocking the expression of caspase-3 can disturb both NLRP3 and GSDME-N expression, but not that of caspase-8. Together, these results suggest that UA induces pyroptosis via the caspase-8/caspase-3/NLRP3/GSDME pathway.

Hyperuricemia is not only associated with cardiovascular diseases (Li L. et al., 2021; Yanai et al., 2021), but also increases the risk of kidney diseases (Obermayr et al., 2008; Bellomo et al., 2010). UA activates the immune system and plays a critical role in inflammation, which is an important pathophysiological mechanism in most kidney diseases (Jung et al., 2020). UA, acting as a damage-associated molecular pattern (DAMP), activates NLRP3 inflammasomes and induces ERK1/2 phosphorylation (Li, 2017; Tao et al., 2019; Yin et al., 2020; Li D. et al., 2021). ERK1/2, which is an abbreviation for extracellular signal-regulated kinases 1 and 2, transmits extracellular signals into the nucleus in response to various stimuli (Ramos, 2008). Our previous study confirmed that TEA inhibited ERK1/2 signal transduction and suppressed the activation of NLRP3 inflammasomes (Li et al., 2019). Our current study confirmed that TEA inhibited UA-induced activation of NLRP3 and phosphorylation of ERK1/2, which was consistent with previous reports.

TEA has been widely used for the treatment of CKD in China. Studies have reported that TEA reduces proteinuria via its anti-inflammatory effect in diabetic nephropathy and adriamycin induced-nephropathy (Tu et al., 2013; Mao et al., 2015). TEA can ameliorate renal tubular epithelial cell injury in streptozotocin (STZ)-induced diabetic nephropathy (DN) mice (Kim et al., 2018), prevent tubulointerstitial fibrosis in chronic renal failure (CRF) rats (Cai et al., 2017) and attenuate tubular cell apoptosis in ischemia/reperfusion (IR)-induced AKI mice (Wu et al., 2019). In a rat model of diabetic nephropathy, TEA ameliorated podocyte apoptosis by inhibiting the expression of caspase-3 and caspase-8. Both podocyte and renal tubular injury contribute to proteinuria. Our previous study has verified that TEA attenuates adriamycin-induced renal tubular injury via activation of the ROS/ERK1/2/NLRP3 signal transduction (Li et al., 2019). In the present study, we confirmed that TEA attenuated UA-induced renal tubular pyroptosis by downregulating caspase-8/caspase-3/NLRP3/GSDME signaling. However, the mechanism by which caspases and gasdermins are involved in kidney damage requires further investigation.

## Conclusion

Collectively, our study is the first to report that caspase-8/caspase-3/NLRP3/GSDME signaling is involved in UA-induced renal tubular cell injury. TEA protects against UA-induced cell injury through its anti-pyroptotic effect. This study provides a basis for the development of novel therapeutic targets and strategies for the treatment of hyperuricemia.

## Data Availability

The original contributions presented in the study are included in the article/Supplementary Material further inquiries can be directed to the corresponding authors.
