# Supplementary material for: Total extract of Abelmoschus manihot L. alleviates uric acid-induced renal tubular epithelial injury via inhibition of caspase-8/caspase-3/NLRP3/GSDME signaling
Source: Front Pharmacol. 2022 Aug 16;13:907980. doi: 10.3389/fphar.2022.907980 (PMC9424722; doi:10.3389/fphar.2022.907980)
Supplement: Supplementary file 1 [file Image1.pdf]

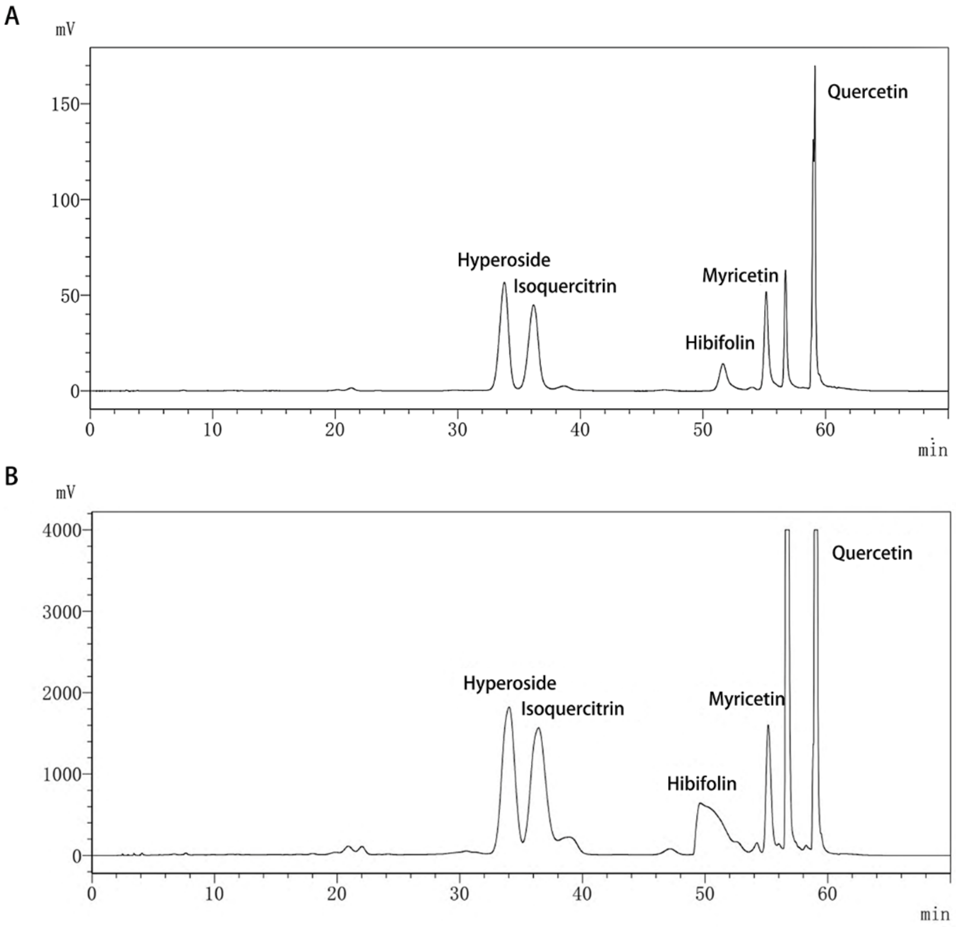

Supplementary Figure 1: Fingerprint analysis of TEA by HPLC. (A) Chromatograms of mixed standards. (B) TEA samples.

Methods of High-Performance Liquid Chromatograph Analysis

Single reference sample solutions of different volumes were measured, mixed, and diluted to produce mixed reference sample solutions containing five components (purchased from Shanghai yuanye Bio-Technology Co., Ltd) as follows: hyperoside (lot no. B20631), isoquercitrin (lot no. B21529), hibifolin (lot no. B29226), myricetin (lot no. B21458) and quercetin (lot no. B20527). The TEA sample was diluted 16 times with distilled water and filtered through a 0.45-μm membrane filter. The Dubhe C18 (250 mm, 4.6 mm, 5 m) chromatographic column with a mobile phase of acetonitrile (A) -0.05% formic acid aqueous solution (B), a gradient elution of: (0-8 min, 15% A, 17% A; 8-45 min, 17% A, 30%A; 45-55 min, 30% A, 80%A; 55–56 min, 80% A, 95% A; 56–57 min, 95% A, 15% A; 57–60 min, 15% A) was used. The velocity was 1.0 ml/min. The detection wavelength was 360 nm. The column temperature was 35°C. The sample quantity was 10 μL.
